# The “Pre-Finishing” Approach in Direct Anterior Restorations. A Case Series

**DOI:** 10.3390/dj9070079

**Published:** 2021-07-07

**Authors:** Gaetano Paolone, Salvatore Scolavino, Enrico Gherlone, Gianrico Spagnuolo, Giuseppe Cantatore

**Affiliations:** 1 Department of Dentistry, IRCCS San Raffaele Hospital and Dental School, Vita Salute University, 20132 Milan, Italy; g.paolone@docenti.unisr.it (G.P.); gherlone.enrico@hsr.it (E.G.); cantatore.giuseppe@hsr.it (G.C.); 2 Independent Researcher, 80035 Nola, Italy; dott.scolavino@gmail.com; 3 Department of Neuroscience, Reproductive and Odontostomatological Sciences, University of Naples “Federico II”, 80131 Naples, Italy

**Keywords:** composite, anterior teeth, direct restoration, layering, incisors, esthetic

## Abstract

In esthetic restorations of anterior teeth the clinician has to manage several aspects in order to have a predictable outcome. A deep knowledge of the anatomy as well as the adhesive procedures and the optical properties of resin-based composites are mandatory to achieve esthetic results. Contemporary restorative materials present either several shades and different translucency properties and therefore they are able to mimic teeth’s optical behavior thus providing a natural aspect to anterior restorations. The wrong thickness of different composite layers may provide unpleasant results such as low value (grayish) restorations that often requires reintervention. A precise step-by-step procedure is therefore mandatory to provide the proper shade at the correct place. There is therefore the need of some corrections and adjustments during the layer procedure in order to avoid errors in shade positioning that could affect final result. The authors present a case series (six clinical cases) treated with the proposed technique with up to five years follow-up.

## 1. Introduction

Resin based composite (RBC) materials show good clinical performance for Class III, IV, shape modification and diastema closure providing excellent results with minimal biological costs [1,2,3,4,5]. Limitations of past resin-based composites like mechanical fracture resistance and surface instability have been partially solved first by micro-hybrid composites and recently with the introduction of nano-hybrid and nano-filled composites. Regardless of mechanical and surface characteristics, various other aspects have improved in RBCs in last decades, such as biocompatibility [6] and cytotoxicity [7,8] optical properties [9]. The evolution of dental composites has allowed in fact the clinician the capability to reproduce either the shade and the translucencies of natural teeth thus providing a natural aspect to anterior restorations [10]. Several layering techniques have been proposed for the restoration of anterior teeth all pointing out the importance of the correct placement of the correct shades/opacities within the restoration [10,11,12,13,14]. Even the smallest misplacement of composite shades and errors in layer thicknesses could in fact result in an esthetic unpleasant outcome [10].

Common errors are generally wrong translucent (enamel) thickness that could result in a low value (grayish) outcome or the revealing of the opaque dentinal layer during the finishing of the restoration [11,15]. These unfavorable clinical situations occur while the clinician loses the correct references during the layering procedure. A precise step-by-step procedure should be followed in order to allow the clinician to always keep a reference to rely on, in order to respect the correct tridimensional placement of the selected shades. There is, therefore, the need of some corrections and adjustments during the layering procedure in order to avoid errors in shade positioning that could affect final result. The authors propose a technique aimed to manage these modifications during the layering procedure. A case series treated with the proposed technique with up to seven years follow-up is presented.

## 2. Case Series Presentation

The proposed technique is described in detail in Section 2.1.2 of the first clinical case. It was applied in all clinical cases presented in the present article.

### 2.1. Case 1

#### 2.1.1. Preliminary Analysis, Isolation, Preparation, Adhesion and Frame Setup

**Figure 1:**
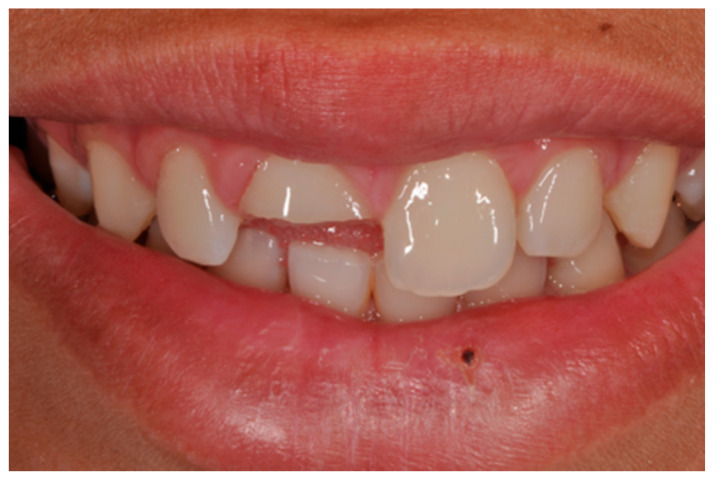
Initial clinical situation. Reprinted from Restauri diretti nei settori anteriori, G. Paolone, S. Scolavino, © 2021, with permission from Quintessence Publishing Italy.

**Figure 2:**
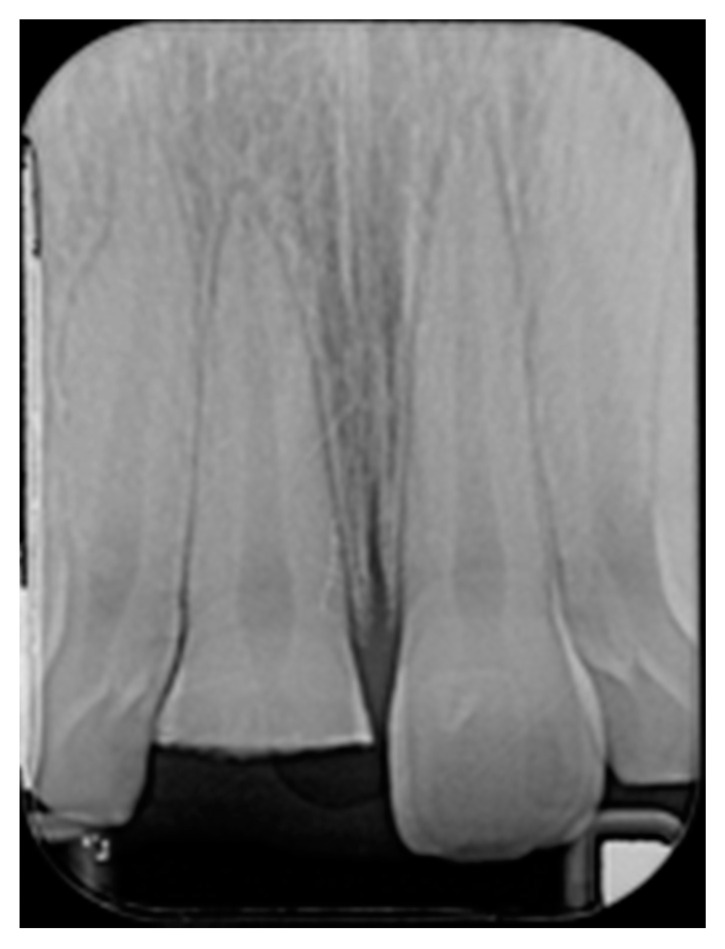
Initial x-ray. Reprinted from Restauri diretti nei settori anteriori, G. Paolone, S. Scolavino, © 2021, with permission from Quintessence Publishing Italy.

**Figure 3:**
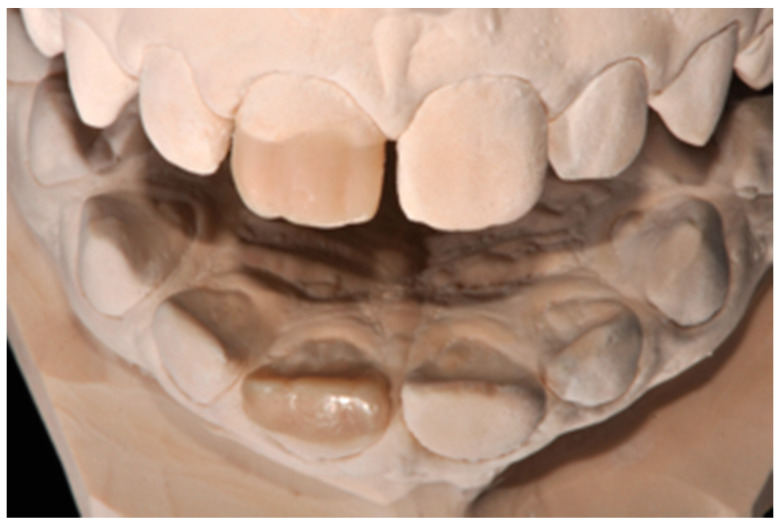
Wax-up. Reprinted from Restauri diretti nei settori anteriori, G. Paolone, S. Scolavino, © 2021, with permission from Quintessence Publishing Italy.

A healthy 10-year boy presented to the dental office after traumatic accident during sport activity. Upper central incisor presented horizontal fracture with no pulp exposure (Figure 1 and Figure 2). Tooth fragment was not retrieved after the traumatic accident. The tooth responded positively to pulp tests (cold and electrical). In order to manage sensitivity and protect the tooth before the restoration appointment, after rubber dam placement, a universal adhesive (Clearfil Universal Bond Quick, Kuraray Noritake Dental, Tokyo, Japan) was applied in self-etch mode followed by a little layer (approximatively 0.5 mm) of flowable composite (Clearfil Majesty ES Flow, A2, Kuraray Noritake Dental, Tokyo, Japan). A silicone impression (Imprint, 3M ESPE, St. Paul, MN, USA) was therefore taken in order to develop a wax-up (Figure 3) and a palatal silicone index (Elite HD+, Zhermack, Badia Polesine, Italy).

**Figure 4:**
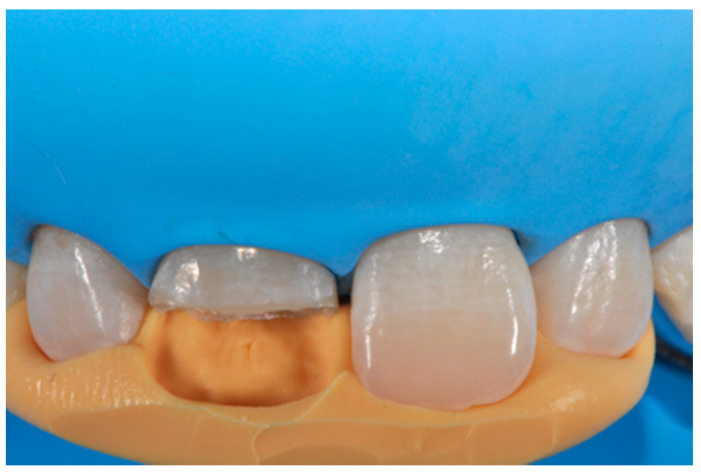
Silicone index try-in after rubber dam isolation. Reprinted from Restauri diretti nei settori anteriori, G. Paolone, S. Scolavino, © 2021, with permission from Quintessence Publishing Italy.

**Figure 5:**
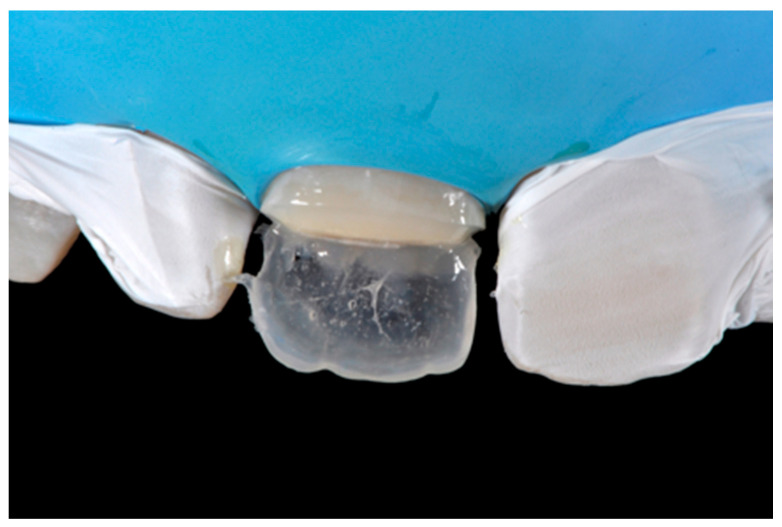
Palatal wall is molded and silicone index removed. Reprinted from Restauri diretti nei settori anteriori, G. Paolone, S. Scolavino, © 2021, with permission from Quintessence Publishing Italy.

**Figure 6:**
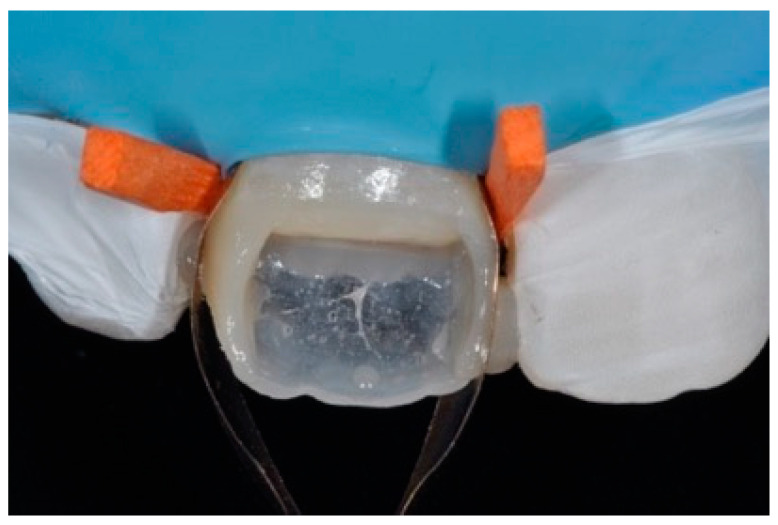
Sectional matrices applied and stabilized with wedges and flowable drops. Reprinted from Restauri diretti nei settori anteriori, G. Paolone, S. Scolavino, © 2021, with permission from Quintessence Publishing Italy.

Before isolation with rubber dam, shade was selected using the button-try technique [14] applying composite samples on the teeth and light-curing them without performing previous adhesive procedures. The silicon index was checked in situ (Figure 4) and interferences were removed with scalpels. A two-step self-etch adhesive system (Clearfil SE Protect, Kuraray Noritake Dental, Tokyo, Japan) was applied following manufacturer’s instructions after selective enamel etching with a 38% phosphoric acid gel (Ultra-Etch, Ultradent Product, Inc., South Jordan, UT, USA). Light curing was applied with a visible light-curing unit with an intensity of 1000 mW/cm^2^ (Valo, Ultradent Products, South Jordan, UT, USA) for 20 s. A thin layer of composite enamel (Clearfil Majesty ES-2, A1E, Kuraray Noritake Dental, Tokyo, Japan) was applied on the silicone index in order to reproduce the palatal wall. The silicone index was then repositioned in the mouth and the palatal composite was adapted to the palatal margin of the preparation and light cured. Another increment was then applied to reproduce the incisal margin. The silicone index was then removed (Figure 5). In order to complete the frame, sectional matrices were applied vertically (Figure 6) and stabilized with wedges and interproximal drops of flowable cured to maintain the desired matrix position (Figure 6). Interproximal walls were then restored using the abovementioned composite enamel.

#### 2.1.2. The ‘Pre-Finishing’ Approach

The technique described in this section consists of three steps and was applied in all clinical cases presented in this article and can be applied to any anterior direct restoration, despite of the materials used.

Step 1 (analysis): Once the frame was completed, a very meticulous analysis was performed on the accuracy of the three components of the frame: palatal wall, incisal margin and interproximal walls. Before placing in fact, the dentinal/opaque material, several aspects must be analyzed about the frame: the correct thickness of the palatal wall, the correct position, design and thickness of the incisal margin, the correct emergence, position, thickness and height of the inter-proximal walls. Every deviation from the ideal thickness, position and emergence of the abovementioned elements could affect the result if not corrected in advance.

Step 2 (modification): The frame therefore is adjusted adding or removing composite material before beginning the internal dentinal body layering.

**Figure 7:**
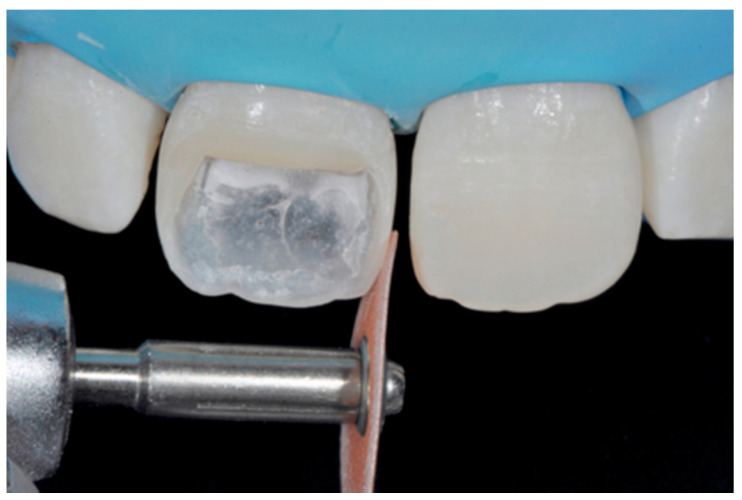
External frame profile is modified with abrasive discs. Reprinted from Restauri diretti nei settori anteriori, G. Paolone, S. Scolavino, © 2021, with permission from Quintessence Publishing Italy.

Excesses external to the frame can be reduced with:

- Burs (WL 268 014 Horico, Berlin, Germany);
- Discs (Sof-Lex, 3M ESPE, St. Paul, MN, USA) (Figure 7);
- Abrasive stripes (Sof-Lex, 3M ESPE, St. Paul, MN, USA);
- EVA points (Proxoshape PS2, Intensive, Montagnola, Switzerland).

**Figure 8:**
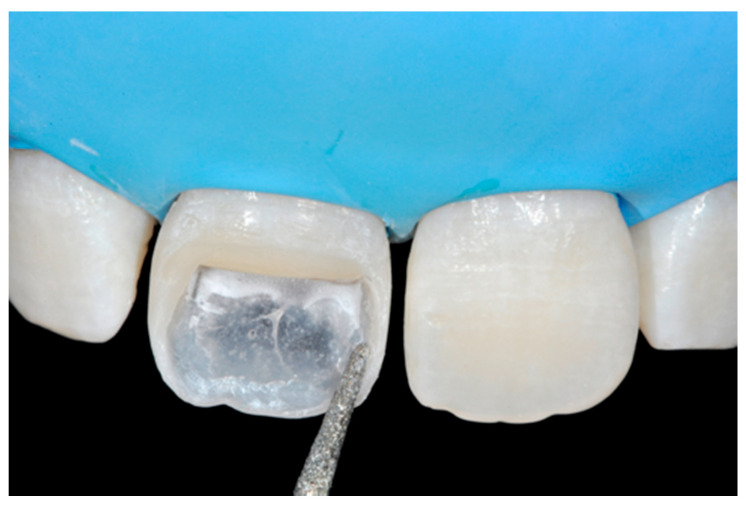
Excessive thickness of interproximal walls is reduced with a low-speed diamond bur. Reprinted from Restauri diretti nei settori anteriori, G. Paolone, S. Scolavino, © 2021, with permission from Quintessence Publishing Italy.

The internal ones can be reduced with diamond burs (WL 268 014 Horico, Berlin, Germany) (Figure 8).

**Figure 9:**
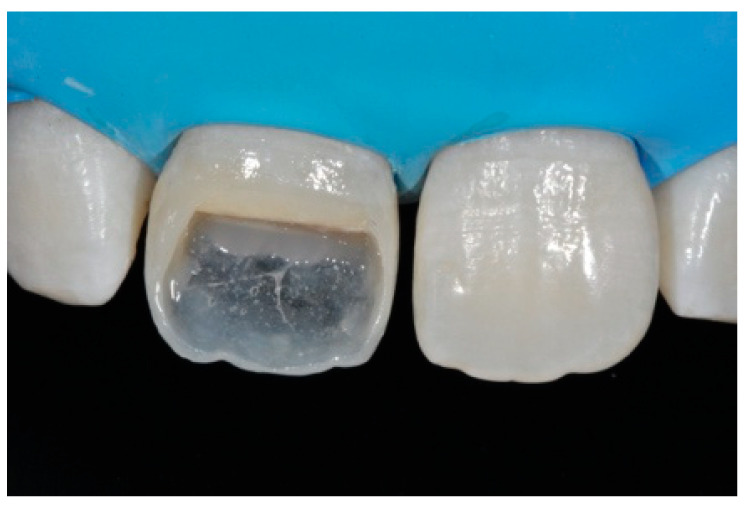
After frame modification silane coupling agent and adhesive procedures are applied. Reprinted from Restauri diretti nei settori anteriori, G. Paolone, S. Scolavino, © 2021, with permission from Quintessence Publishing Italy.

Step 3 (adhesive procedure): After frame modification, dust was removed with strong air pressure and internal surface was treated with a silane coupling agent (Monobond Plus, Ivoclar Vivadent, Schaan, Liechtenstein) applied with a microbrush in a thin layer and allowed to react for 60 s. An adhesive (OptiBond FL, Kerr, Bioggio, Switzerland) was applied following manufacturer’s instructions, and photoactivated for 20 s (Figure 9).

#### 2.1.3. Completion of the Layering Procedure, Finishing and Polishing

**Figure 10:**
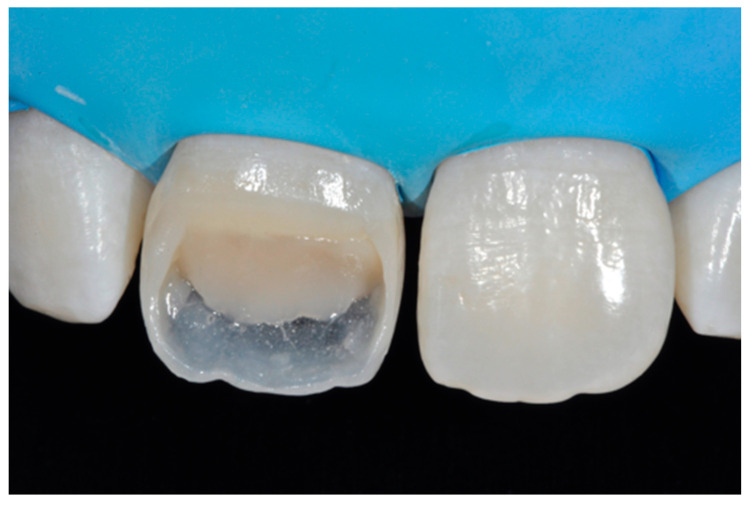
First increment of the dentinal body. Reprinted from Restauri diretti nei settori anteriori, G. Paolone, S. Scolavino, © 2021, with permission from Quintessence Publishing Italy.

**Figure 11:**
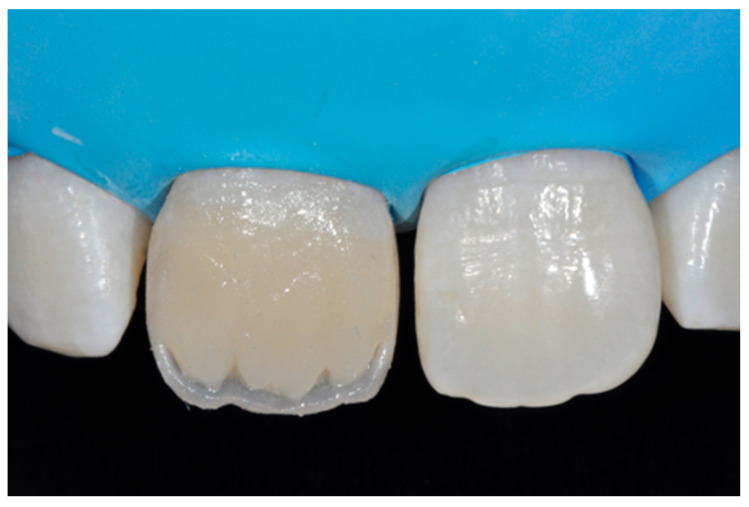
Dentinal body completed. Reprinted from Restauri diretti nei settori anteriori, G. Paolone, S. Scolavino, © 2021, with permission from Quintessence Publishing Italy.

**Figure 12:**
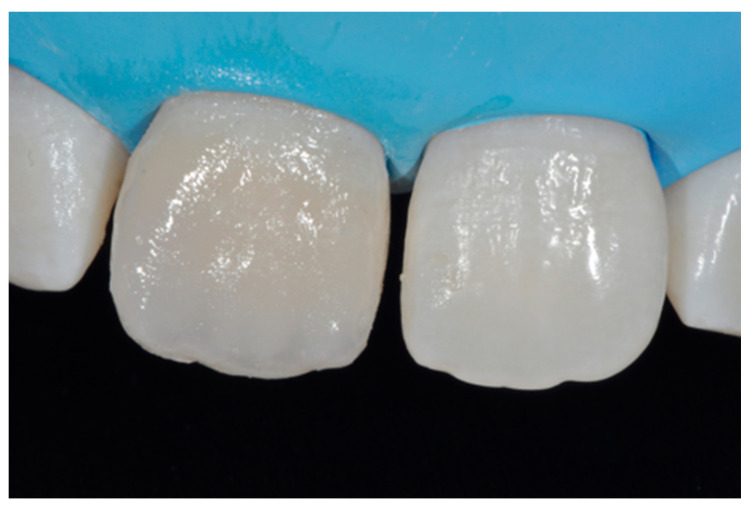
Enamel layer applied. Reprinted from Restauri diretti nei settori anteriori, G. Paolone, S. Scolavino, © 2021, with permission from Quintessence Publishing Italy.

A single shade of dentin (Clearfil Majesty ES-2, A1D, Kuraray Noritake Dental, Tokyo, Japan) was then applied placing separate increments (Figure 10 and Figure 11) to build the dentinal body, leaving space for incisal translucencies. The translucent shade (Clearfil Majesty ES-2, A1E, Kuraray Noritake Dental, Tokyo, Japan) was then applied to complete the restoration (Figure 12).

**Figure 13:**
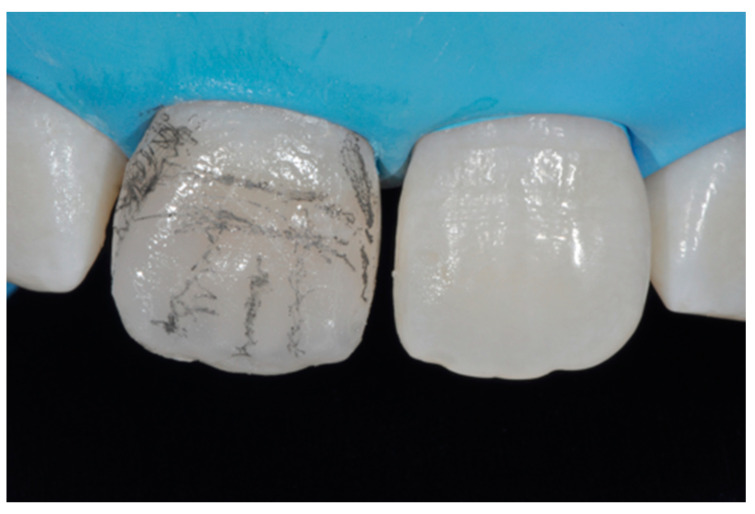
Vertical and horizontal anatomy outlined with a pencil. Reprinted from Restauri diretti nei settori anteriori, G. Paolone, S. Scolavino, © 2021, with permission from Quintessence Publishing Italy.

**Figure 14:**
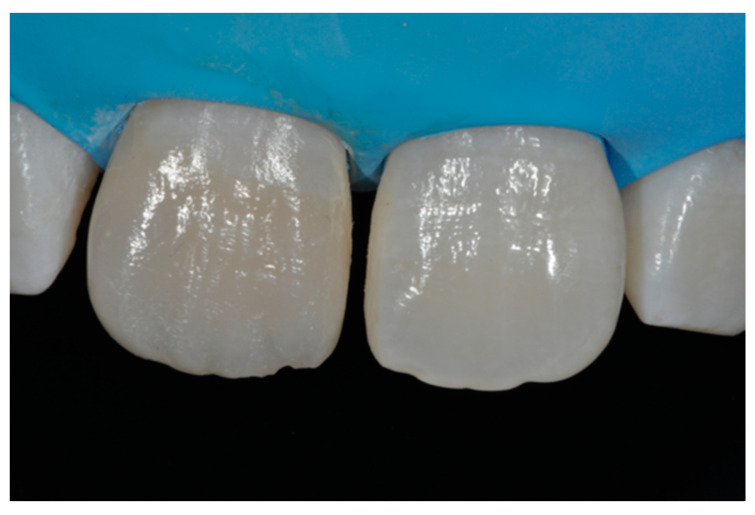
After vertical and horizontal anatomy definition and final polishing. Reprinted from Restauri diretti nei settori anteriori, G. Paolone, S. Scolavino, © 2021, with permission from Quintessence Publishing Italy.

**Figure 15:**
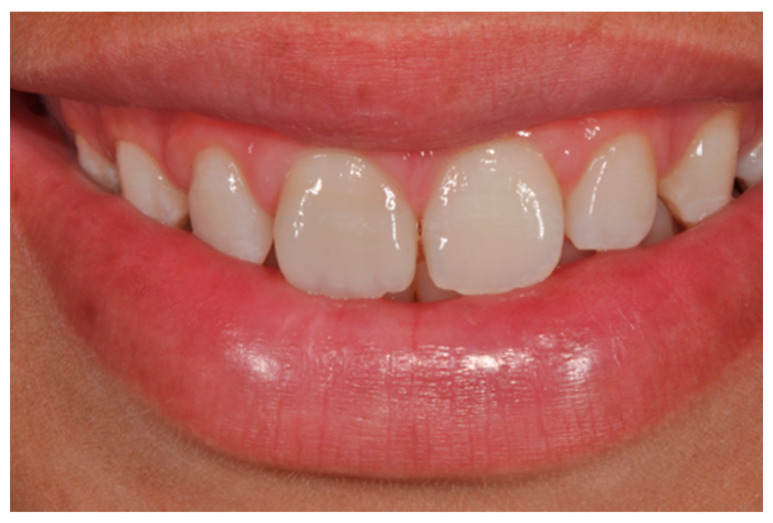
Three months post-operative. Reprinted from Restauri diretti nei settori anteriori, G. Paolone, S. Scolavino, © 2021, with permission from Quintessence Publishing Italy.

**Figure 16:**
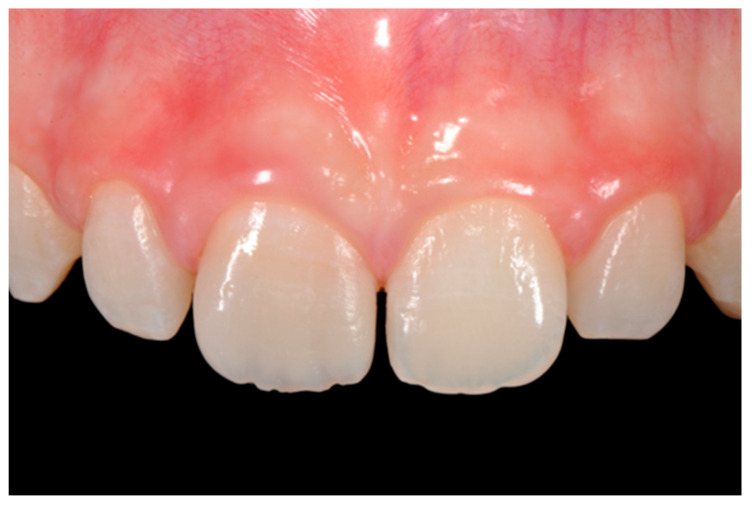
One year post-operative. Reprinted from Restauri diretti nei settori anteriori, G. Paolone, S. Scolavino, © 2021, with permission from Quintessence Publishing Italy.

**Figure 17:**
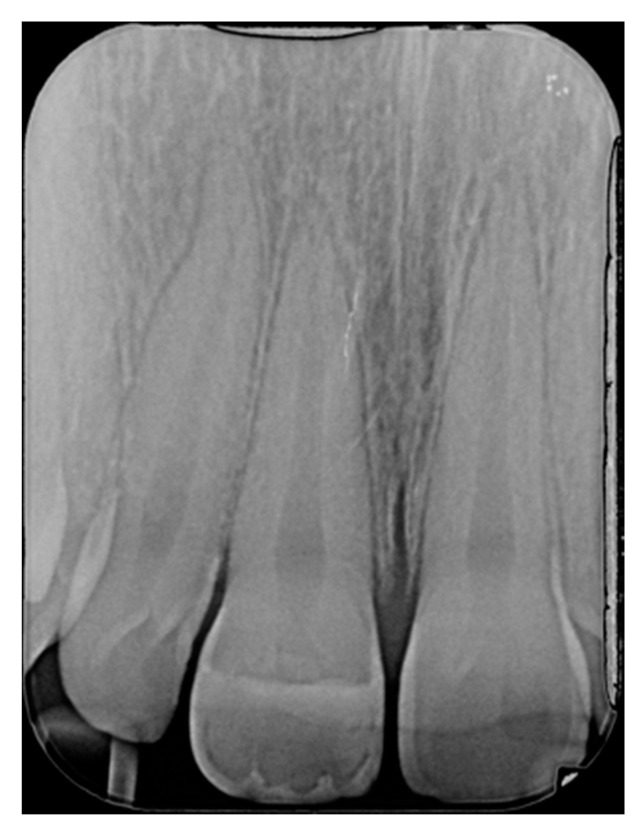
One year post-operative x-ray. Reprinted from Restauri diretti nei settori anteriori, G. Paolone, S. Scolavino, © 2021, with permission from Quintessence Publishing Italy.

**Figure 18:**
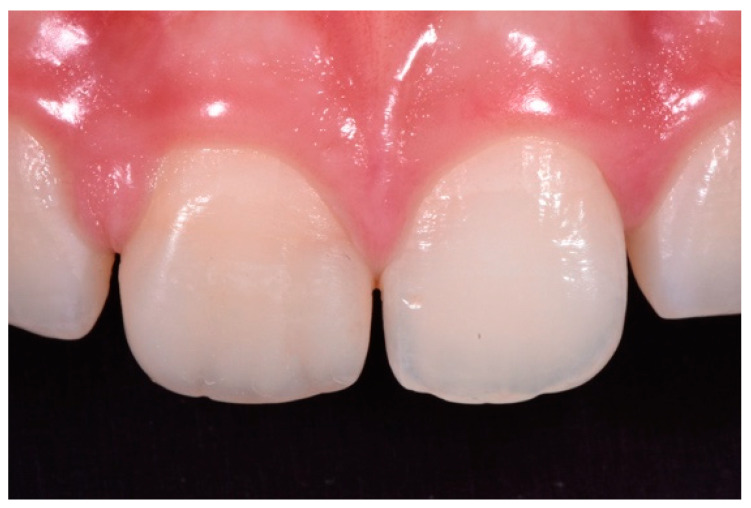
Five years post-operative.

**Figure 19:**
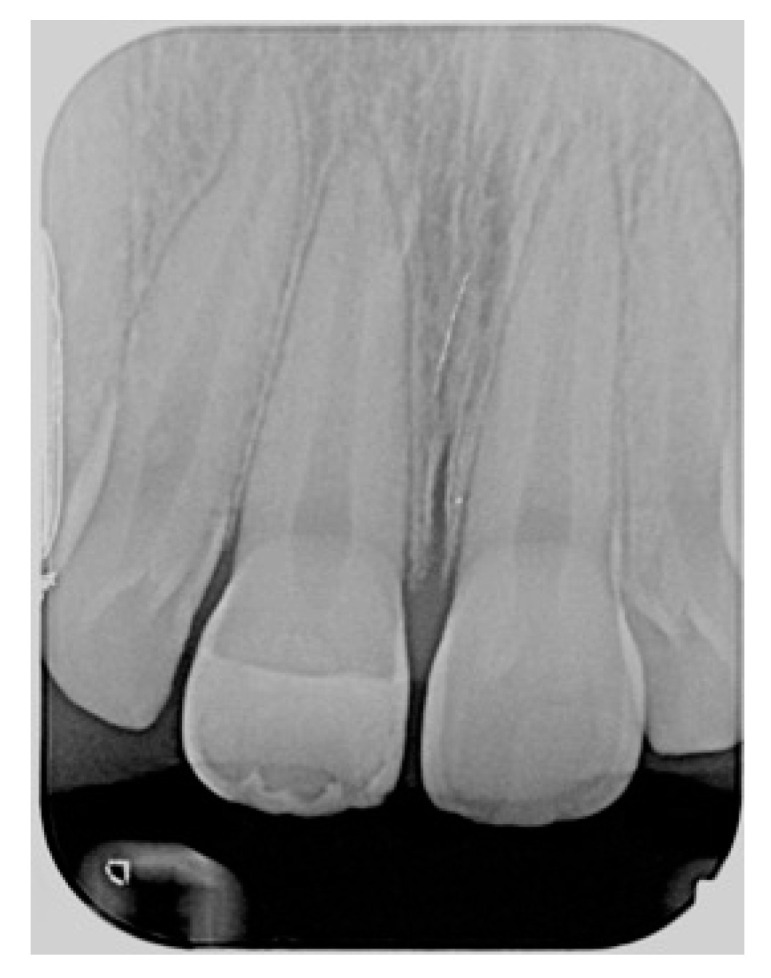
Five years post-operative x-ray.

Finishing and polishing procedures were performed with a diamond bur (WL 268 014 Horico, Berlin, Germany), silicone points (Identoflex, Kerr, Bioggio, Switzerland) brushes (Jiffy Goat Air Brushes, Ultradent Products, South Jordan, UT, USA), and diamond pastes (Diamond Polish Mint, Ultradent Products, South Jordan, UT, USA) (Figure 13 and Figure 14). Satisfactory clinical and radiographic outcome was considered satisfactory at 3-months, 1-year, and 5-years post-operative (Figure 15, Figure 16, Figure 17, Figure 18 and Figure 19).

### 2.2. Case 2

**Figure 20:**
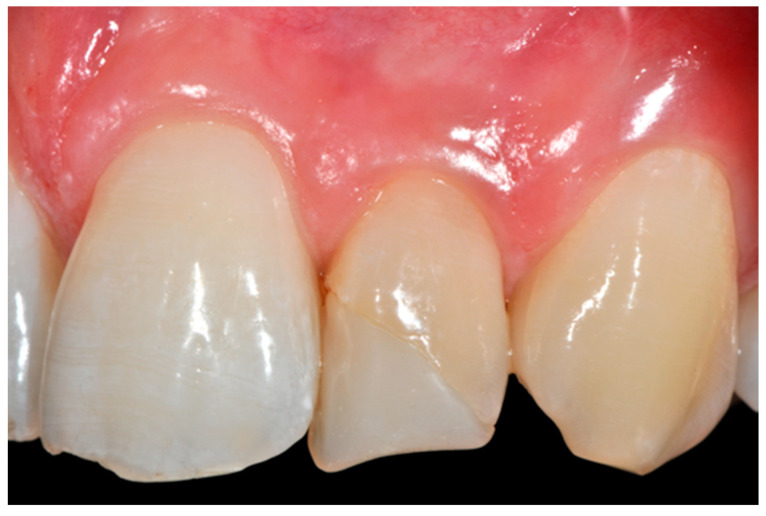
Initial clinical situation. Reprinted from Restauri diretti nei settori anteriori, G. Paolone, S. Scolavino, © 2021, with permission from Quintessence Publishing Italy.

**Figure 21:**
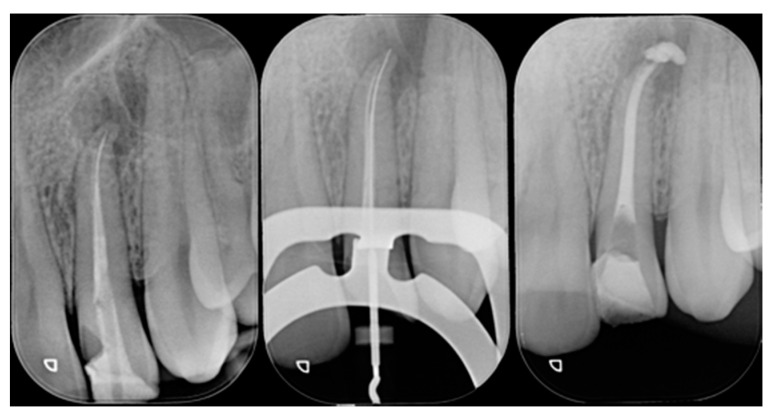
Initial x-ray, during and after endodontic retreatment. Reprinted from Restauri diretti nei settori anteriori, G. Paolone, S. Scolavino, © 2021, with permission from Quintessence Publishing Italy.

**Figure 22:**
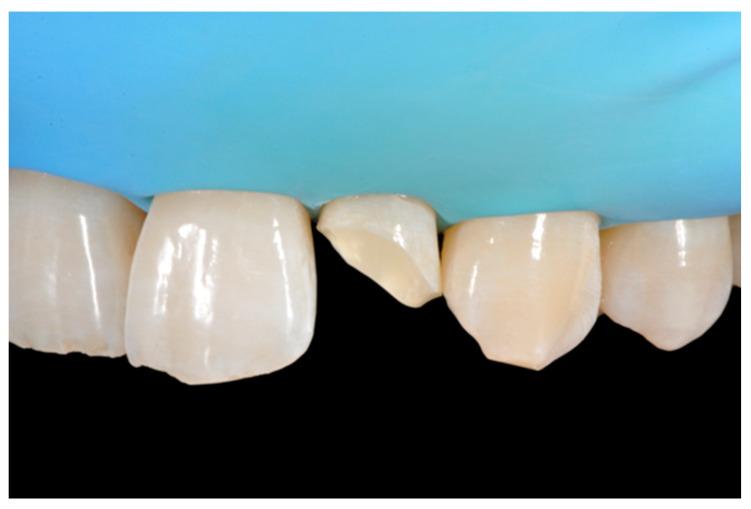
After isolation preparation is performed. Reprinted from Restauri diretti nei settori anteriori, G. Paolone, S. Scolavino, © 2021, with permission from Quintessence Publishing Italy.

**Figure 23:**
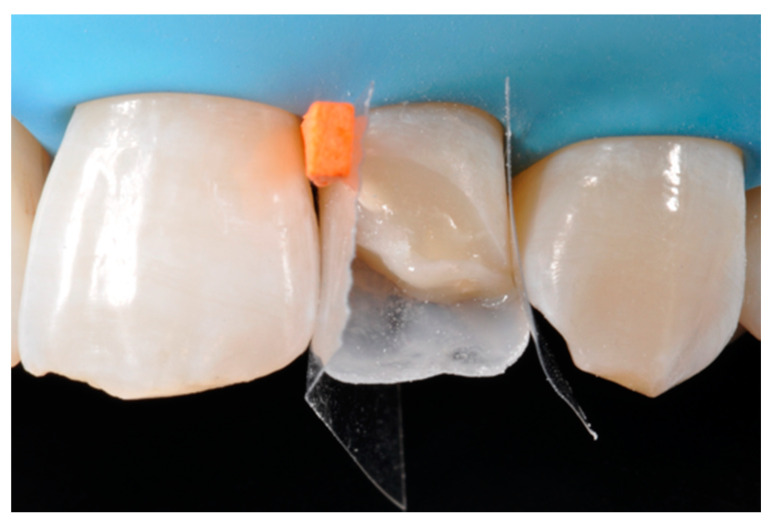
Frame is completed with interproximal matrices. Reprinted from Restauri diretti nei settori anteriori, G. Paolone, S. Scolavino, © 2021, with permission from Quintessence Publishing Italy.

**Figure 24:**
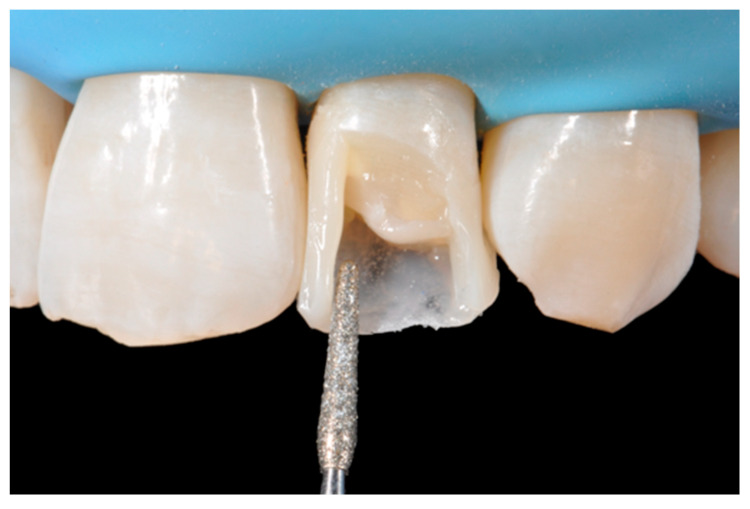
Frame is corrected with the help of diamond burs. Reprinted from Restauri diretti nei settori anteriori, G. Paolone, S. Scolavino, © 2021, with permission from Quintessence Publishing Italy.

**Figure 25:**
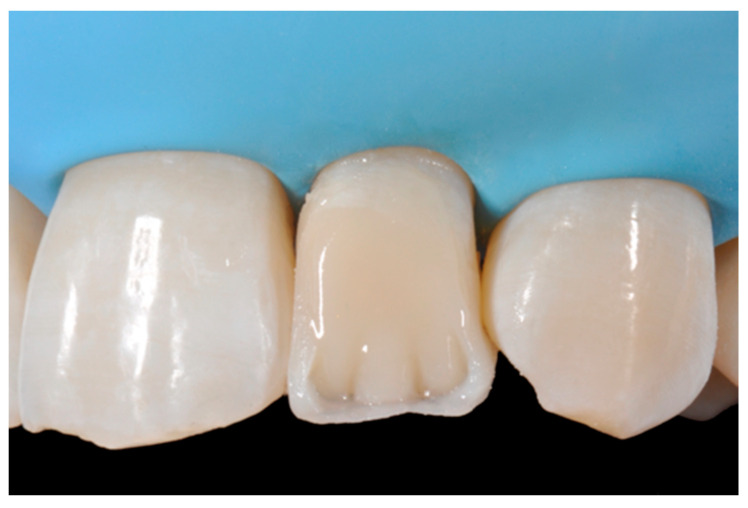
Internal dentinal anatomy. Reprinted from Restauri diretti nei settori anteriori, G. Paolone, S. Scolavino, © 2021, with permission from Quintessence Publishing Italy.

**Figure 26:**
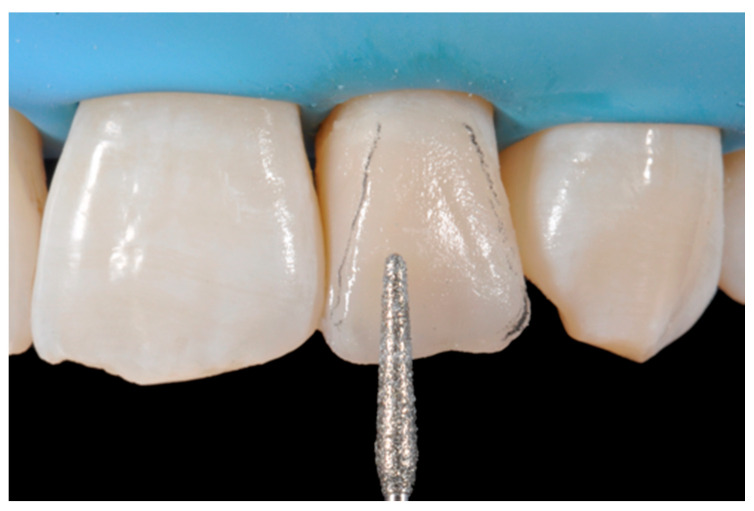
Finishing is performed with discs and diamond burs. Reprinted from Restauri diretti nei settori anteriori, G. Paolone, S. Scolavino, © 2021, with permission from Quintessence Publishing Italy.

**Figure 27:**
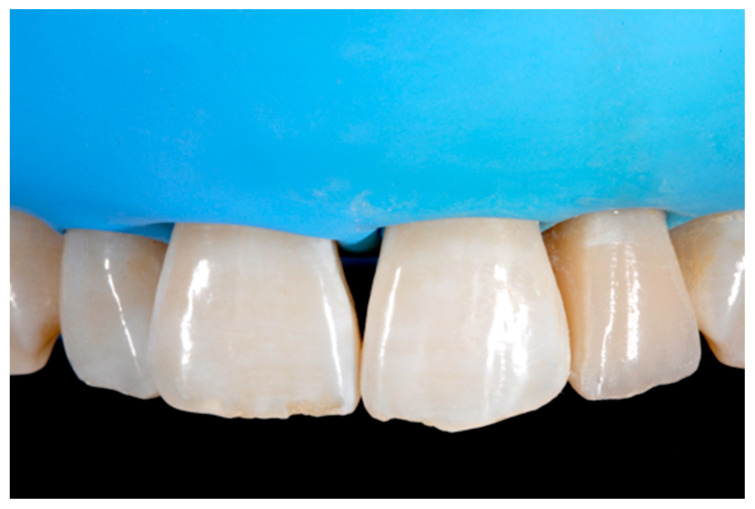
Clinical situation after polishing procedures. Reprinted from Restauri diretti nei settori anteriori, G. Paolone, S. Scolavino, © 2021, with permission from Quintessence Publishing Italy.

**Figure 28:**
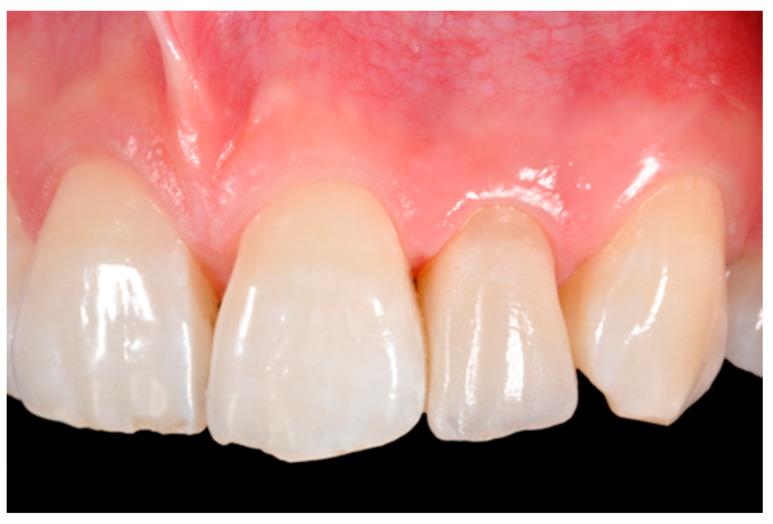
Six-months post-operative. Reprinted from Restauri diretti nei settori anteriori, G. Paolone, S. Scolavino, © 2021, with permission from Quintessence Publishing Italy.

**Figure 29:**
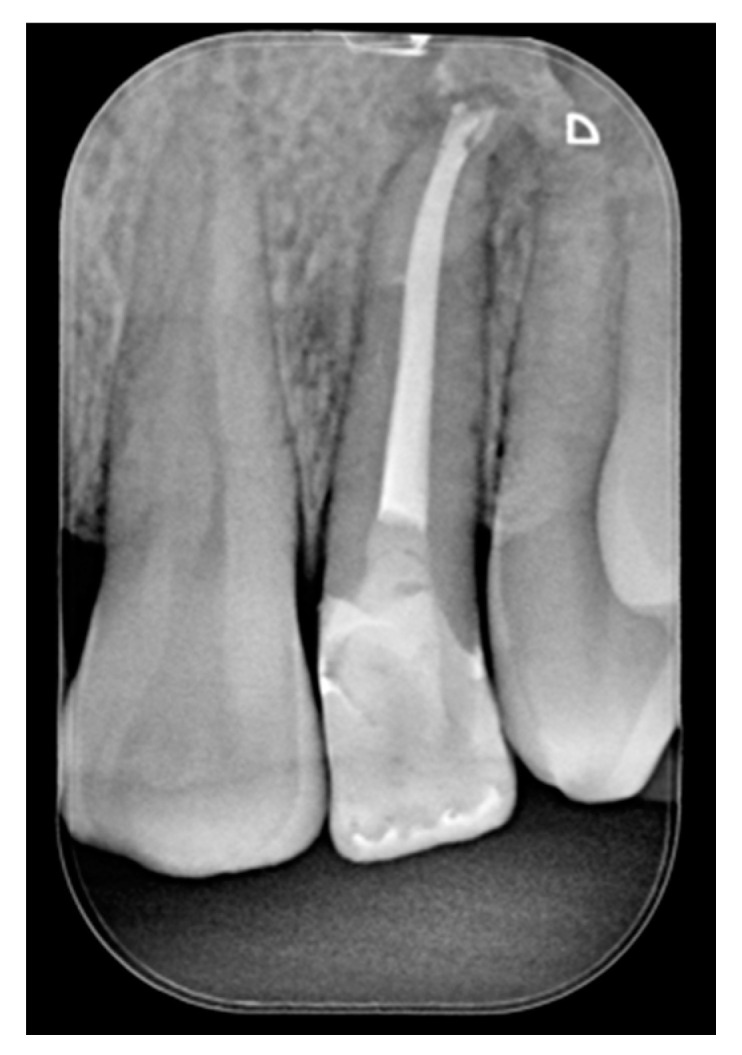
Six-months post-operative x-ray. Reprinted from Restauri diretti nei settori anteriori, G. Paolone, S. Scolavino, © 2021, with permission from Quintessence Publishing Italy.

A 42-year old woman presented to the clinic with esthetic concerns related to her upper left lateral incisor (Figure 20). Pre-operative x-rays showed periapical lesion that required retreatment. After retreatment (Figure 21) a treatment plan was proposed to the patient. The ideal treatment would have involved an orthodontic therapy to gain back lost space in mesial portion. The patient decided not to consider an orthodontic treatment and accepted just the restorative proposal (Class IV restoration). Considered the sufficient amount of ferrule no post was applied [16]. Shade was selected as described in previous case, using the button-try technique [14]. After isolation with rubber dam, preparation (Figure 22) and adhesion (Clearfil SE, Kuraray Noritake Dental, Tokyo, Japan), composite frame was completed (Clearfil Majesty ES-2, A1D, Kuraray Noritake Dental, Tokyo, Japan) with silicone index and interproximal transparent mylar matrices (Figure 23). Interproximal walls were considered too thick; therefore, they were reduced (Figure 24) using diamond bur as described in Section 2.1.2. This allowed the internal dentinal body to have the correct volume (Figure 25) and therefore to respect ideal opacity/translucency ratios. Restoration was therefore completed adding enamel layer (Clearfil Majesty ES-2, A1E, Kuraray Noritake Dental, Tokyo, Japan) and performing finishing and polishing procedures (Figure 26 and Figure 27) as described in previous clinical case. The restoration shows good integration 6 months post-operative (Figure 28 and Figure 29).

### 2.3. Case 3

**Figure 30:**
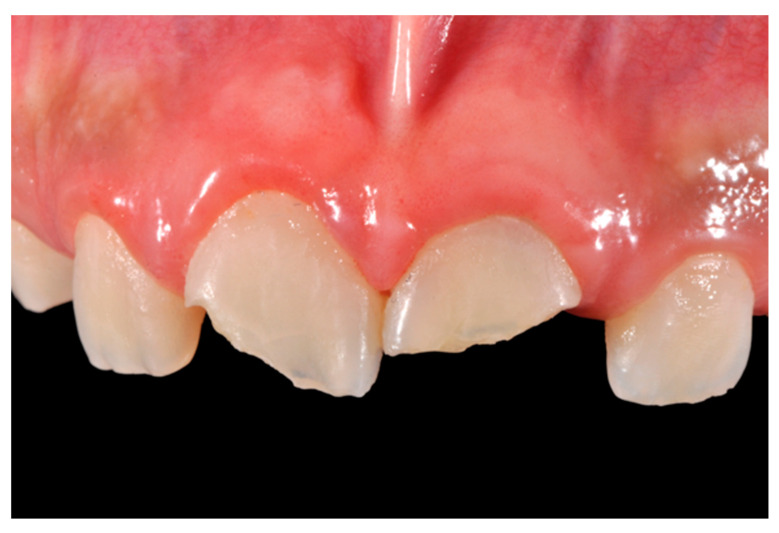
Initial clinical situation. Reprinted from Restauri diretti nei settori anteriori, G. Paolone, S. Scolavino, © 2021, with permission from Quintessence Publishing Italy.

**Figure 31:**
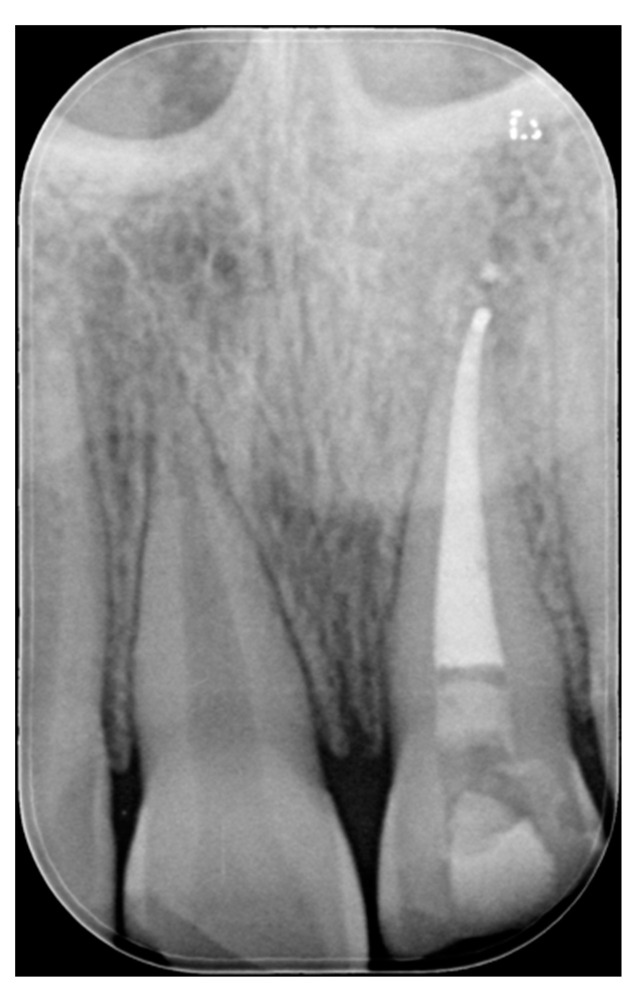
X-ray after endodontic treatment. Reprinted from Restauri diretti nei settori anteriori, G. Paolone, S. Scolavino, © 2021, with permission from Quintessence Publishing Italy.

**Figure 32:**
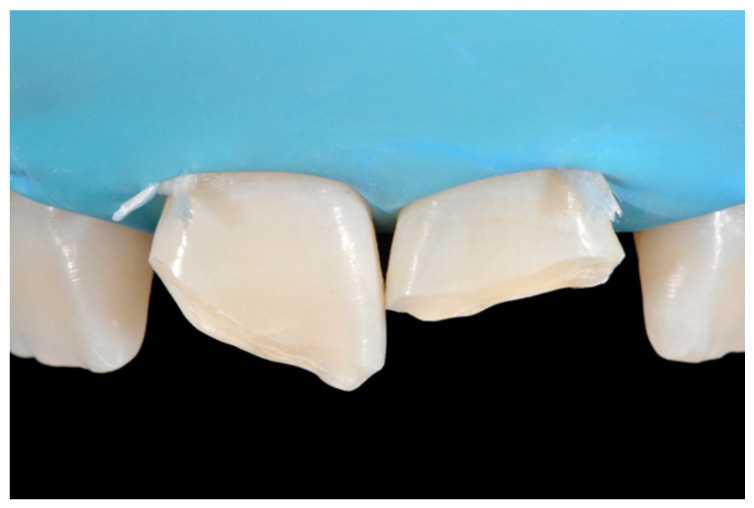
Isolation and preparation. Reprinted from Restauri diretti nei settori anteriori, G. Paolone, S. Scolavino, © 2021, with permission from Quintessence Publishing Italy.

A 27-years lady presented to the dental office after traumatic accident (Figure 30). Tooth #1.1 responded positively to pulp tests (cold and electrical) while Tooth #2.1 showed pulp exposure and continuous bleeding. Endodontic treatment was therefore performed on #2.1 (Figure 31) and direct restoration of both teeth was planned. Shade was selected as described in previous case, using the button-try technique [14]. Even in this clinical case, considered the sufficient ferrule, no post was applied on Tooth #2.1. After isolation and preparation (Figure 32), adhesive procedures were performed using a self-etch two-step adhesive with selective etching procedure (Clearfil SE, Kuraray Noritake Dental, Tokyo, Japan).

**Figure 33:**
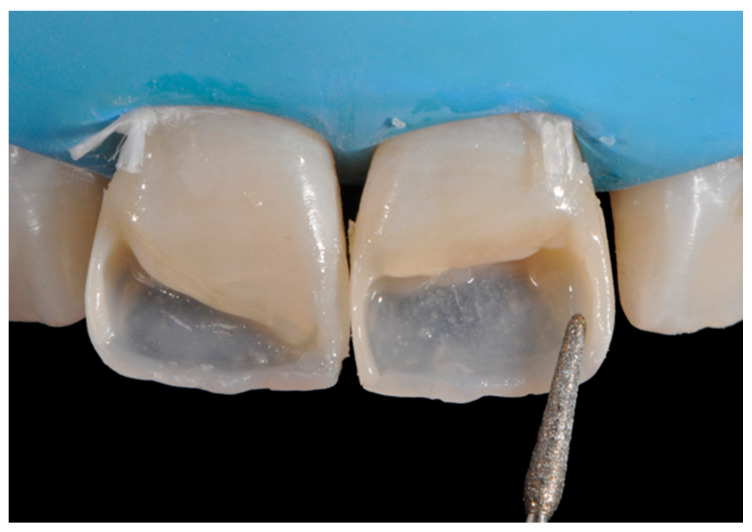
Frame imprecisions are corrected by reducing interproximal wall from the internal side. Reprinted from Restauri diretti nei settori anteriori, G. Paolone, S. Scolavino, © 2021, with permission from Quintessence Publishing Italy.

**Figure 34:**
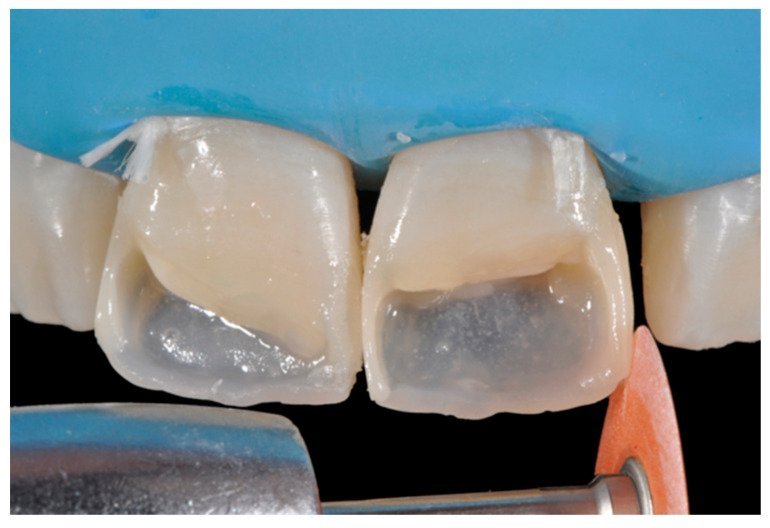
Frame is also trimmed reducing external outline. Reprinted from Restauri diretti nei settori anteriori, G. Paolone, S. Scolavino, © 2021, with permission from Quintessence Publishing Italy.

**Figure 35:**
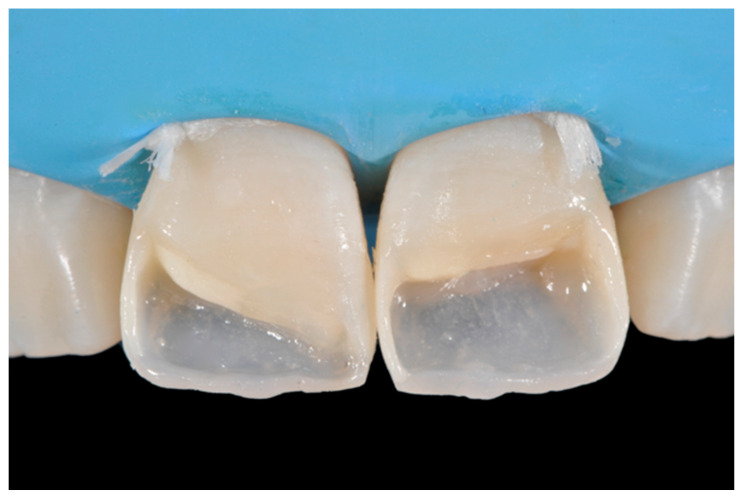
The modified frames treated with silane coupling agent and adhesive, ready for layering of dentinal body. Reprinted from Restauri diretti nei settori anteriori, G. Paolone, S. Scolavino, © 2021, with permission from Quintessence Publishing Italy.

**Figure 36:**
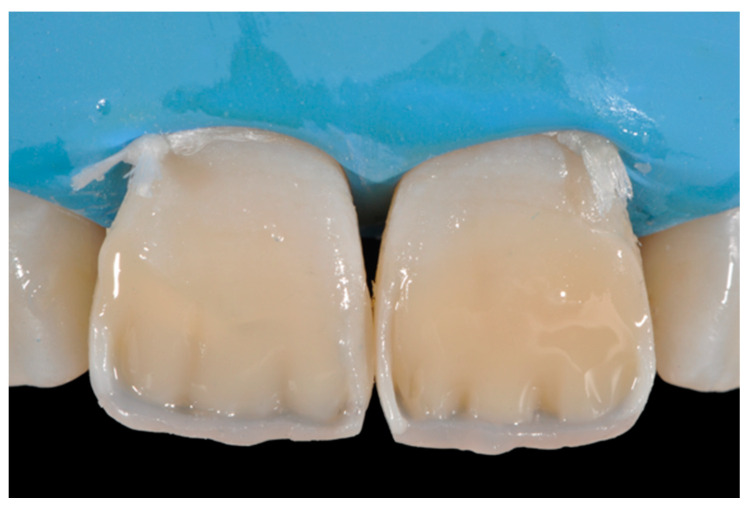
The dentinal body. Reprinted from Restauri diretti nei settori anteriori, G. Paolone, S. Scolavino, © 2021, with permission from Quintessence Publishing Italy.

**Figure 37:**
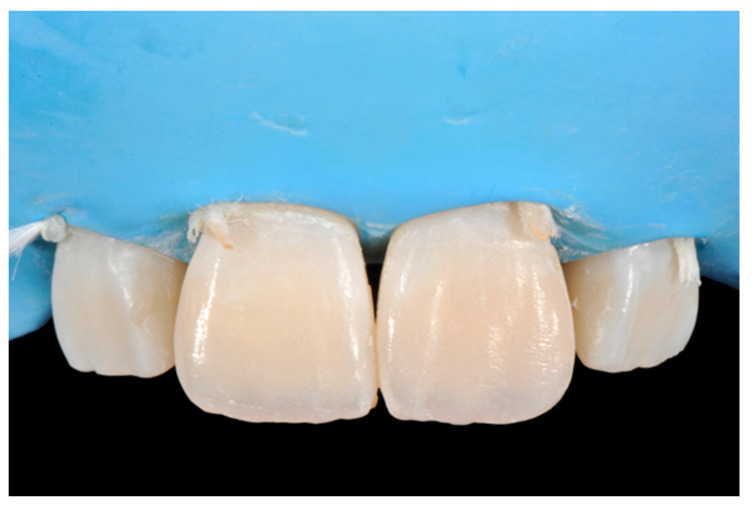
Final result after finishing and polishing. Reprinted from Restauri diretti nei settori anteriori, G. Paolone, S. Scolavino, © 2021, with permission from Quintessence Publishing Italy.

**Figure 38:**
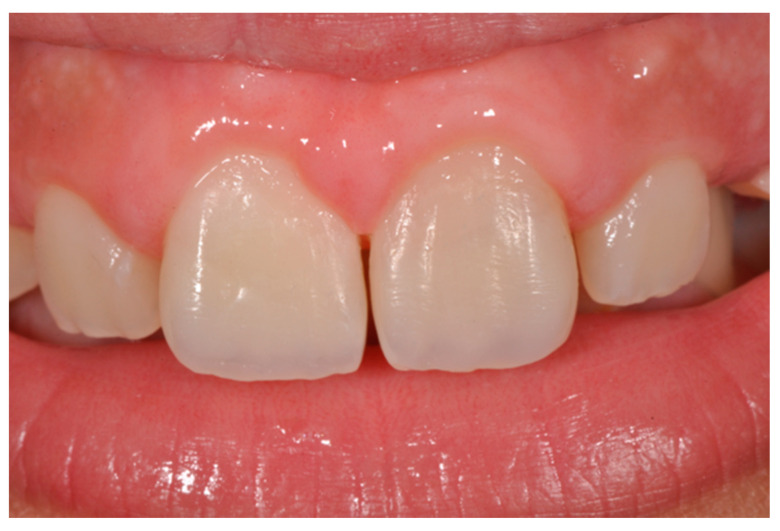
**At** 1.5 years, post-operative clinical view. Reprinted from Restauri diretti nei settori anteriori, G. Paolone, S. Scolavino, © 2021, with permission from Quintessence Publishing Italy.

**Figure 39:**
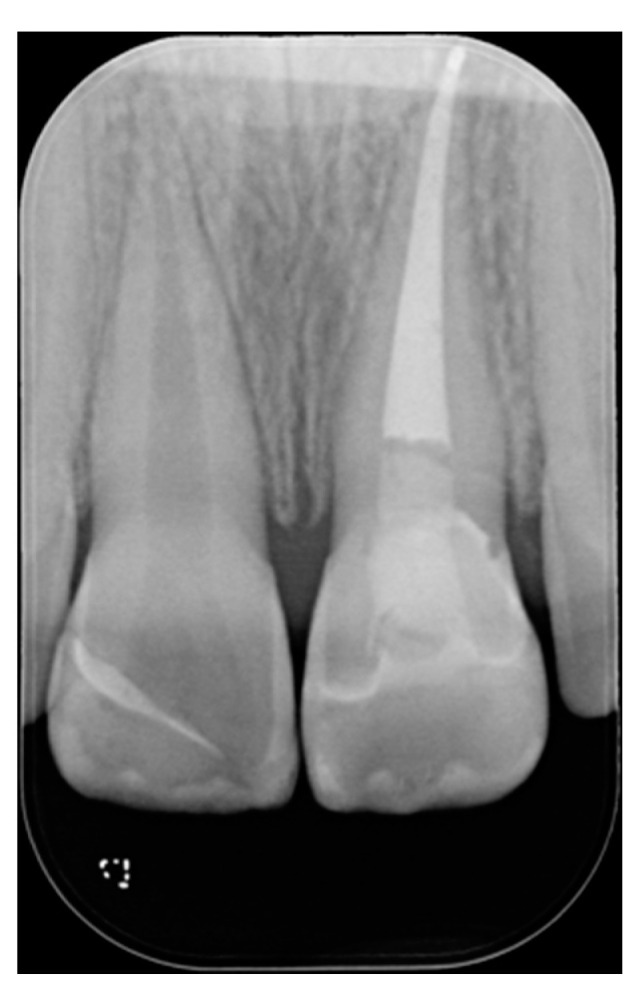
At 1.5 years, post-operative radiographic view. Reprinted from Restauri diretti nei settori anteriori, G. Paolone, S. Scolavino, © 2021, with permission from Quintessence Publishing Italy.

Once completed, the two frames (Clearfil Majesty ES-2, A1E, A2D, Kuraray Noritake Dental, Tokyo, Japan) showed internal and external excesses that were reduced using diamond burs and discs (Figure 33 and Figure 34) strictly following the procedure described in Section 2.1.2 of present article. Excesses were removed from the distal-incisal angle of #2.1 allowing therefore to obtain the desired translucency. After silane application and bonding procedure (Figure 35) as described in Section 2.1.2, restorations were completed (Figure 36 and Figure 37). They both show satisfactory clinical integration 1.5 years post-operative (Figure 38 and Figure 39).

### 2.4. Case 4

**Figure 40:**
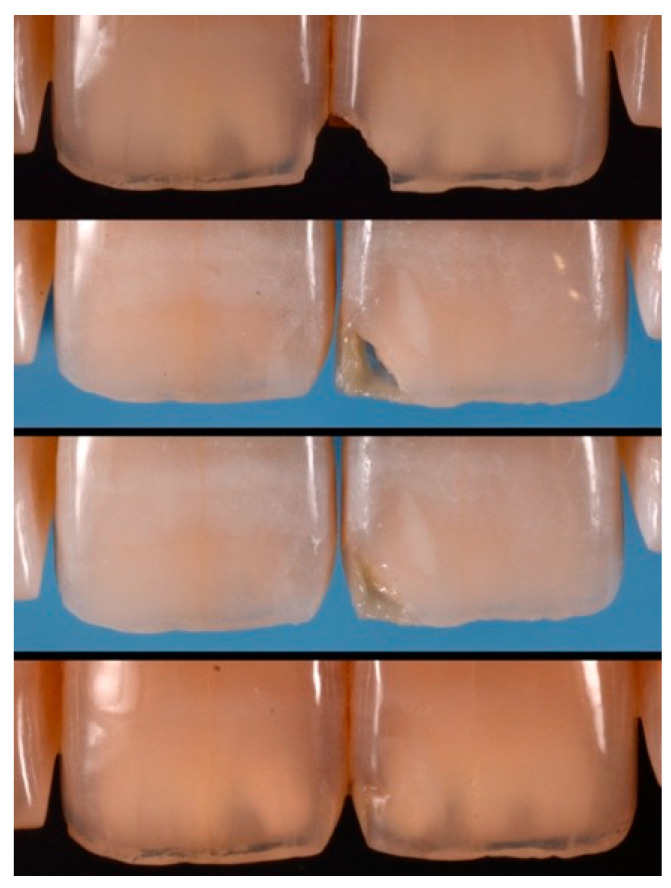
Pre-operative, restorative procedures and post-operative of #1.1 and #2.1. Reprinted from Restauri diretti nei settori anteriori, G. Paolone, S. Scolavino, © 2021, with permission from Quintessence Publishing Italy.

A 53-years old man presented to the dental office asking for the restoration of the mesial incisal angles of both central incisors (Figure 40). After preliminary shade analysis (using the button-try technique [14]) an attentive management of opaque and translucent shades was required to esthetically solve this case. After isolation with rubber dam, preparation, and adhesive procedures (Optibond FL, Kerr, Bioggio, Switzerland) the frame was completed using a translucent shade (Mosaic, ET, Ultradent Products, South Jordan, UT, USA) for palatal wall and an opaque material (Mosaic, A2, Ultradent Products, South Jordan, UT, USA) for incisal margin and interproximal walls. The external and internal excesses of the frame were modified (as described in Section 2.1.2) to obtain a uniform thin opaque outline as planned during the preliminary color/opacity analysis. This modification was performed to avoid the unfavorable uncovering of translucent areas during final finishing procedures that could result in an unaesthetic outcome. Once the definition of the external frame was completed, the mesial mamelon was modeled (Mosaic, A2, Ultradent Products, South Jordan, UT, USA) and the translucent material (Mosaic, ET, Ultradent Products, South Jordan, UT, USA) was applied. Finishing and polishing procedures (Jiffy, Ultradent Products, South Jordan, UT, USA) were completed afterwards.

### 2.5. Case 5

**Figure 41:**
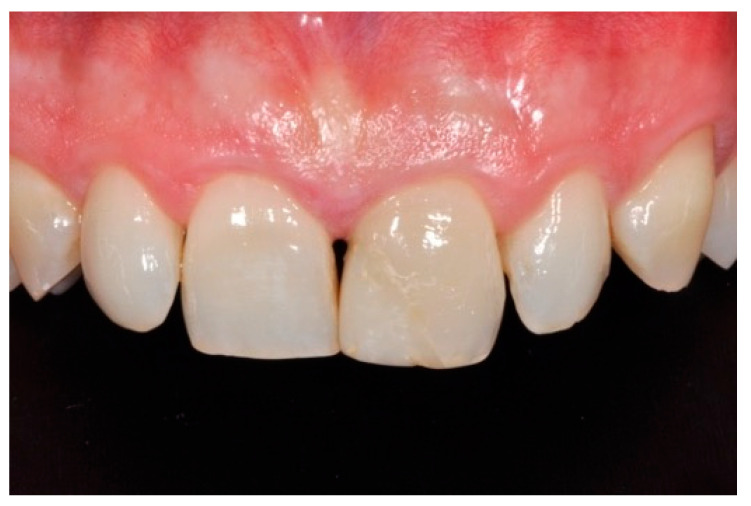
Initial clinical situation.

**Figure 42:**
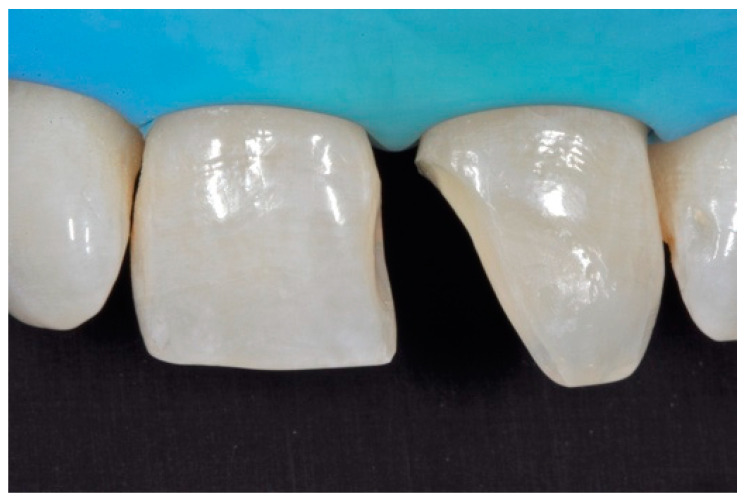
Preparation of Class III on #1.1 and Class IV on #2.1.

**Figure 43:**
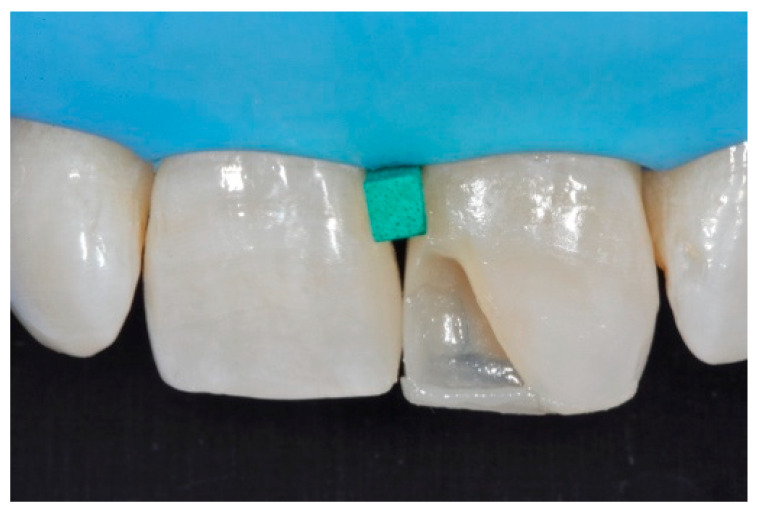
The frame completed.

**Figure 44:**
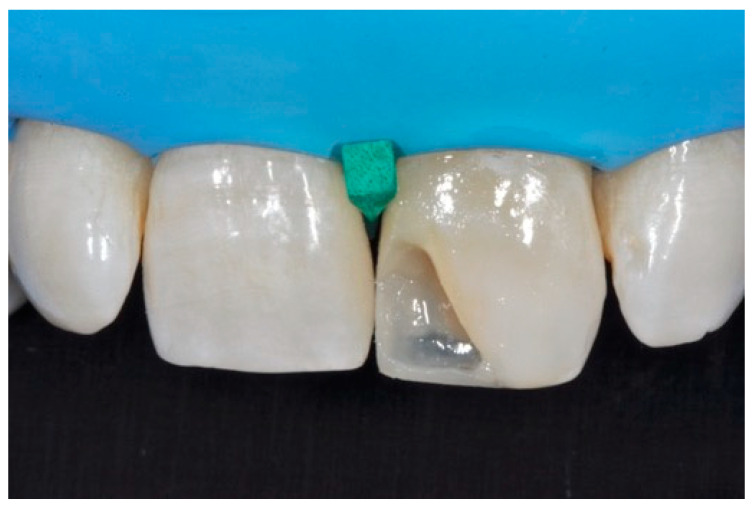
The frame modified either in the incisal frame and in the interproximal wall.

**Figure 45:**
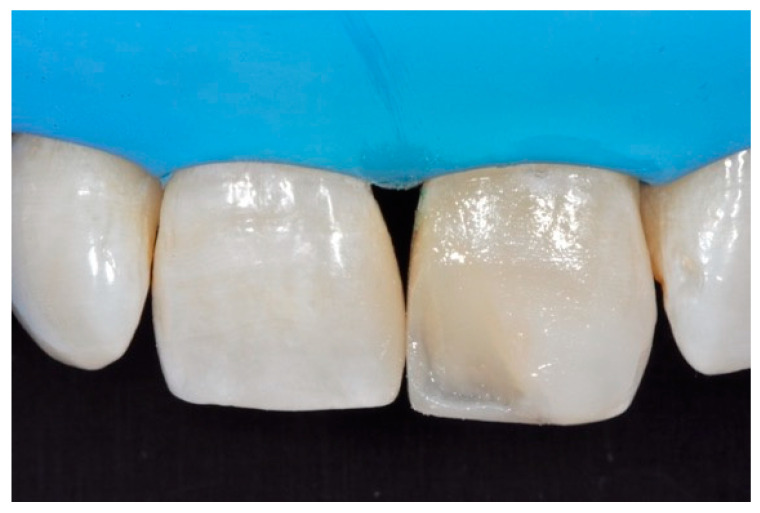
Dentinal body with space left for translucencies.

**Figure 46:**
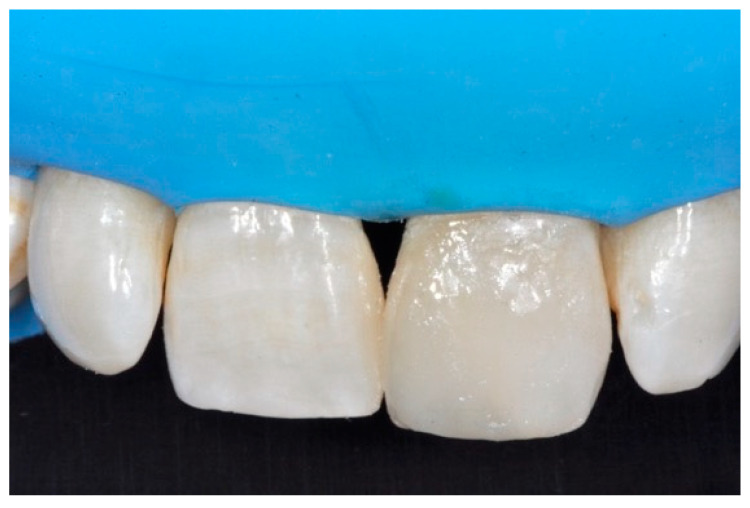
External enamel layer is applied.

**Figure 47:**
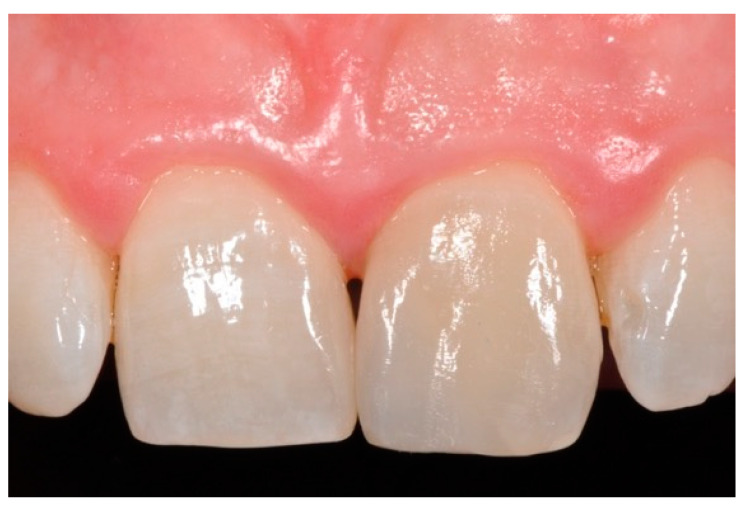
Six months post-operative.

A healthy 38-year-old man referred to the dental office for the esthetic rehabilitation of left central maxillary incisor (Figure 41). Shade was selected as described in previous cases, using the button-try technique [14]. Isolation, preparation (Figure 42) and adhesive procedures were performed (Tokuyama Bond Force, Tokuyama Dental, Osaka, Japan). and Class III was restored on right central incisor (Asteria, A2B, Tokuyama Dental, Osaka, Japan). Frame was then completed on #2.1 (Figure 43) with the use of posterior sectional matrices using translucent and body material (Asteria, NE, A2B Tokuyama Dental, Osaka, Japan). After removing excesses both from the incisal margin and from the interproximal portion (Figure 44) silane and adhesive was applied strictly following the procedure described in Section 2.1.2. Dentinal body (Asteria, A2B, Tokuyama Dental, Osaka, Japan) was applied to reproduce internal anatomy (Figure 45) and then the external translucent enamel (Figure 46) (Asteria, NE, Tokuyama Dental, Osaka, Japan) was applied. The restoration shows good integration 6-months post-operative (Figure 47).

### 2.6. Case 6

**Figure 48:**
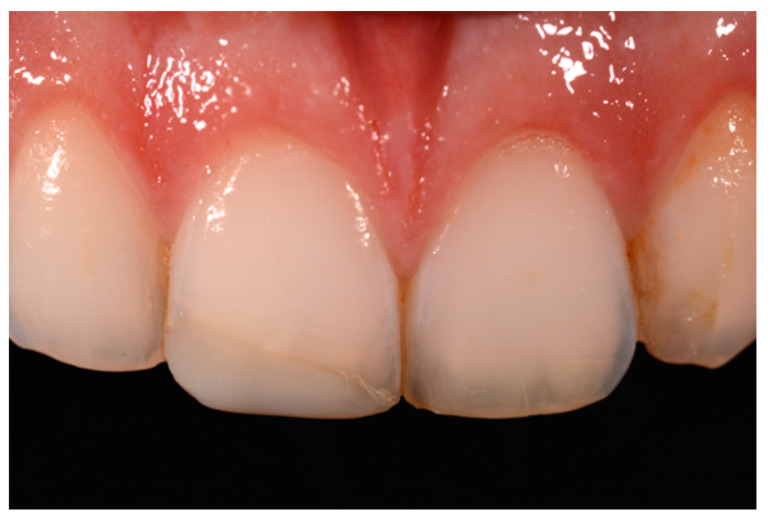
Initial clinical situation.

**Figure 49:**
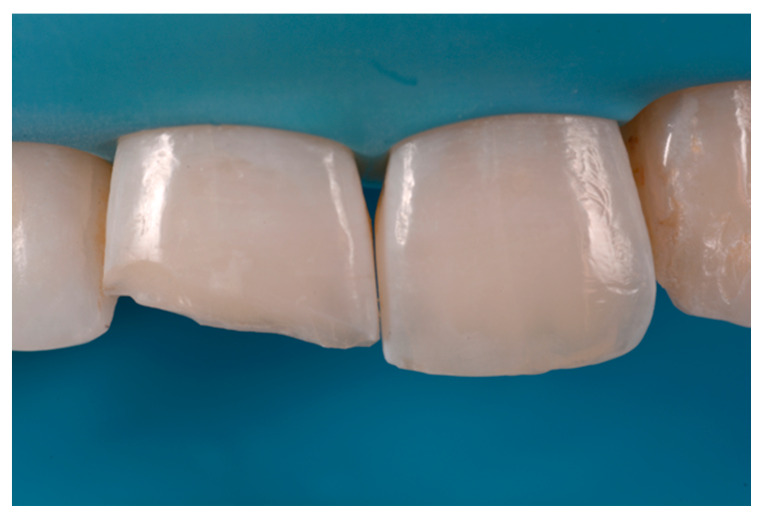
Margin preparation.

**Figure 50:**
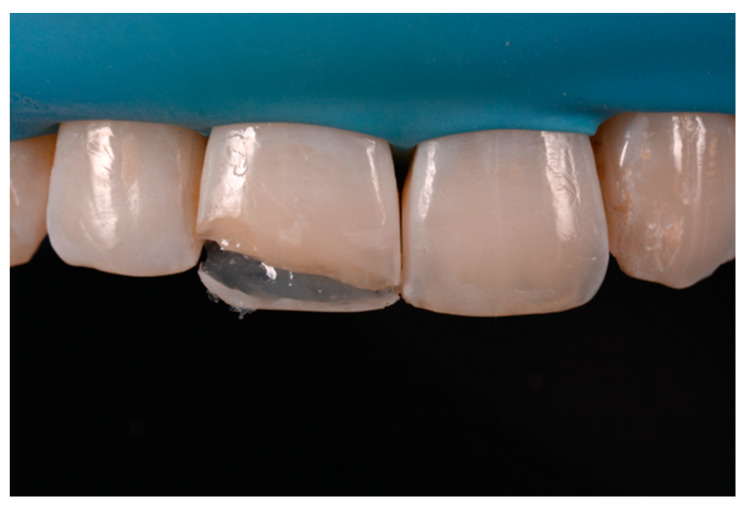
Palatal translucent and incisal opaque material molded using a silicone index.

**Figure 51:**
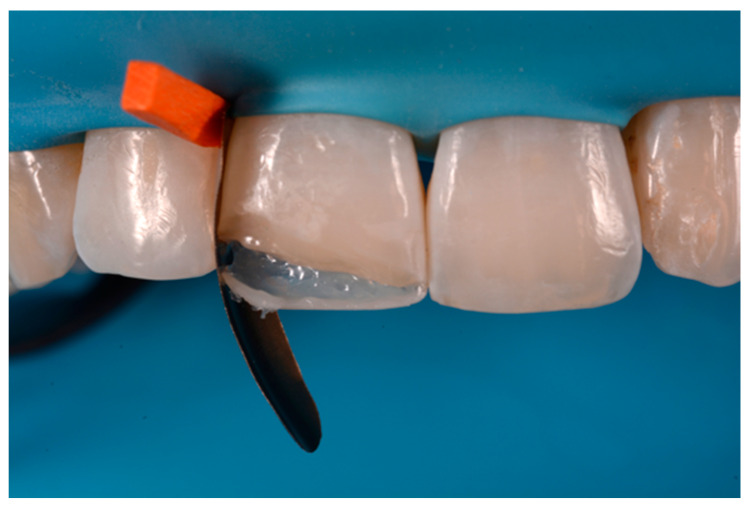
Sectional matrix applied to restore the distal wall.

**Figure 52:**
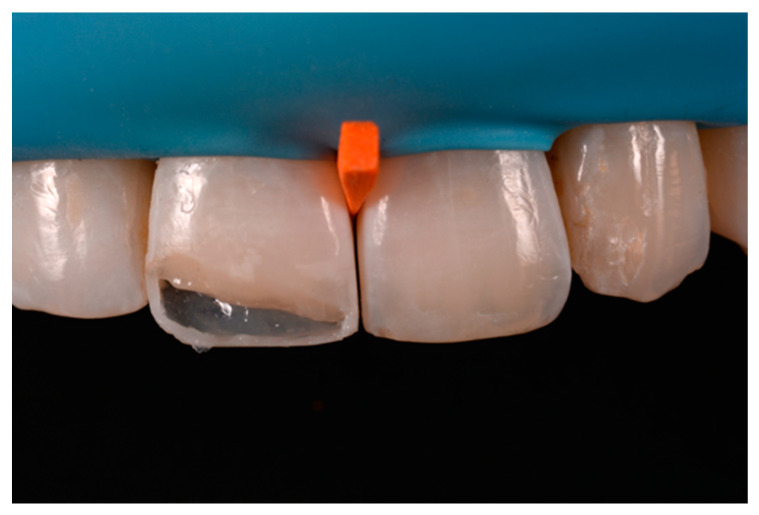
The frame completed.

A healthy 23-year-old man referred to the dental office for the esthetic rehabilitation of right central maxillary incisor (Figure 48). Shade was selected as described in previous cases, using the button-try technique [14]. After isolation, preparation (Figure 49) and adhesive procedures (Prime & Bond Active, Dentsply Sirona, York, PA, USA), the frame was completed (Figure 50, Figure 51 and Figure 52) using a silicone index prepared upon a wax-up using translucent and opaque materials (Ceram.x Spectra Effects D1, E1, A1, Dentsply Sirona, York, PA, USA).

**Figure 53:**
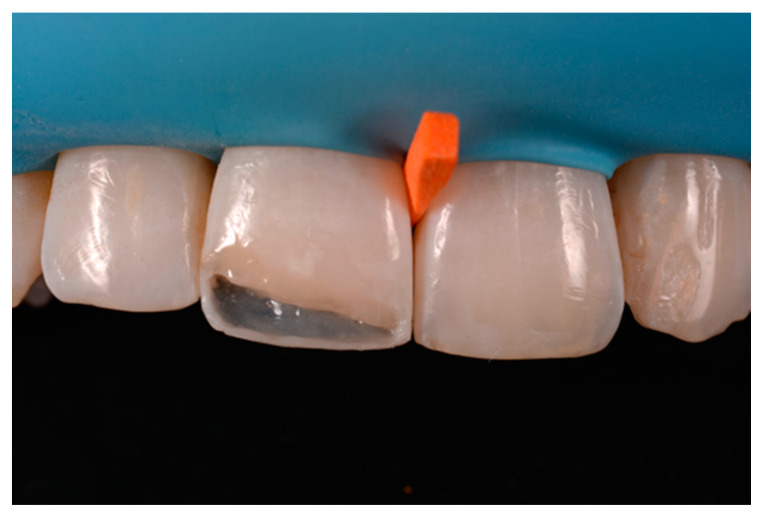
After frame is modified, silane coupling agent and adhesive is applied before dentinal body layering as described in Section 2.1.2.

**Figure 54:**
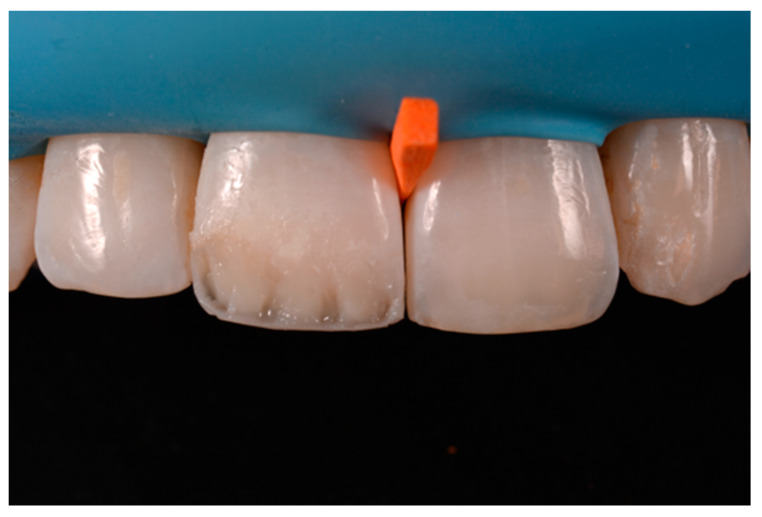
Dentinal body.

**Figure 55:**
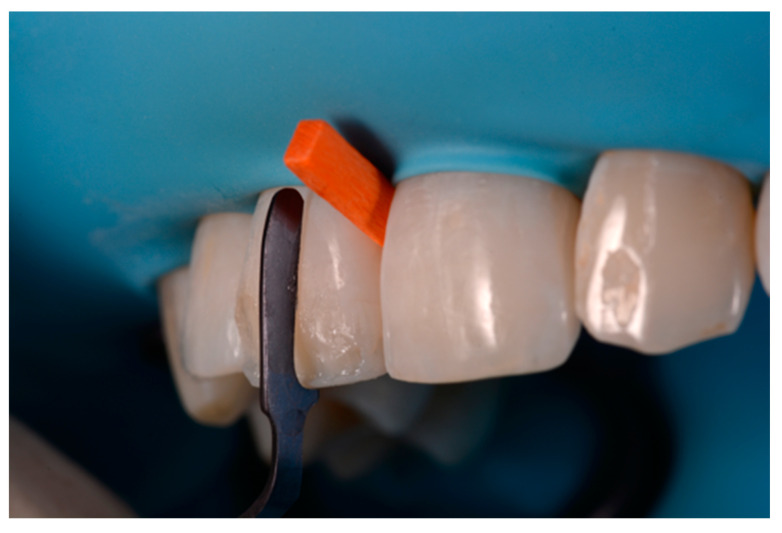
Checking and modeling external translucent shade with specific caliper.

**Figure 56:**
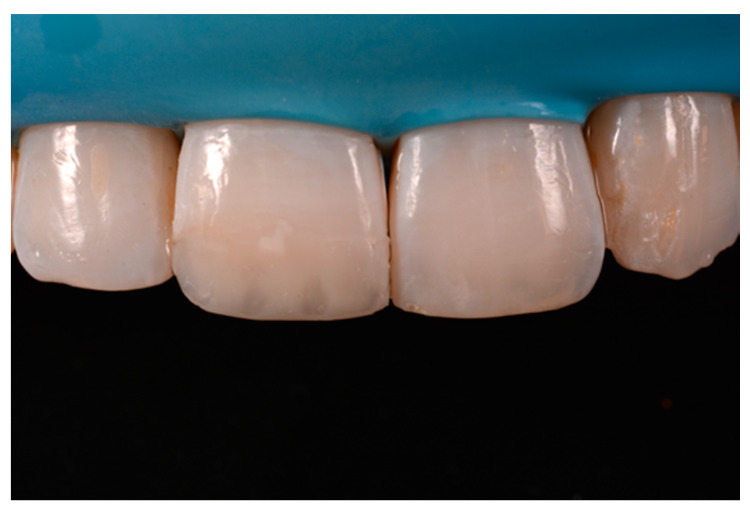
Translucent shade is applied, finishing and polishing performed.

**Figure 57:**
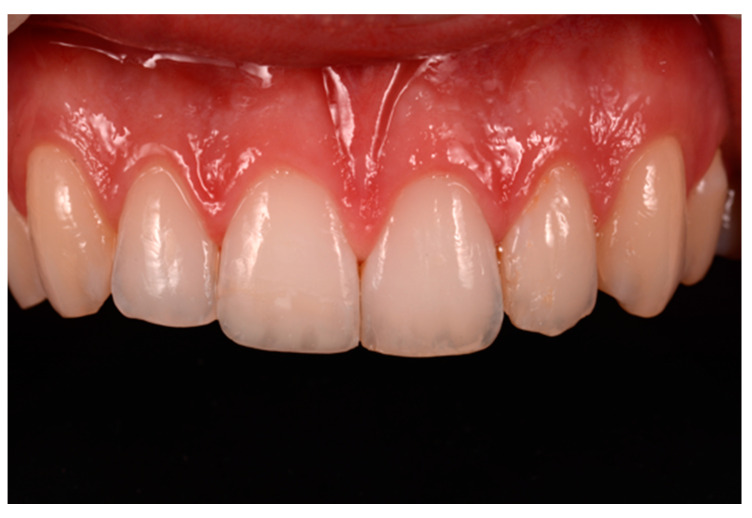
One-year post-operative.

Excesses were removed from the frame (Figure 53) and silane and adhesive were applied following the step-by-step procedure described in Section 2.1.2. Dentinal body (Ceram.x Spectra Effects D1, Dentsply Sirona, York, PA, USA) (Figure 54) was applied to reproduce internal anatomy and then the external translucent enamel and body (Ceram.x Spectra Effects E1, A1, Dentsply Sirona, York, PA, USA) were applied checking precisely thickness and removing excesses with a caliper (Figure 55 and Figure 56) (TNCALIBRA, HuFriedy, Chicago, IL, USA). The restoration shows good integration one-year post-operative (Figure 57).

## 3. Discussion

Esthetic direct restorations in anterior teeth show median overall success rate of 95% after 10 years for Class III and of 90% for Class IV [17]. In respect to posterior direct restorations, esthetic factors play a fundamental role in in anterior restorations’ success rate. Baldissera et al. reported in fact higher failures for esthetic reasons in respect to other failures (fracture) for anterior resin-based composite (RBC) restorations [18]. It is, therefore, mandatory for the clinician to provide predictable esthetic results in their restorative procedures in anterior region. The final esthetic outcome of an RBC restoration depends on several factors such as the material’s properties [19,20,21], the operator’s skill level [22], the adhesive procedure [23], the shade selection [24], the correct composite opacity/translucency selection [10], the quality of the substrate [15] and the surface management [25].

Natural teeth are characterized both from shades and from opaque and translucent areas. Contemporary composites can emulate both characteristics, providing therefore, the clinician powerful tools to face esthetic challenges. The preliminary color analysis of a tooth generally involves a basic shade selection and the detection of opaque and translucent areas [15]. Commercially available composites can in fact provide more opaque materials, generally used for internal dentinal anatomy or for opaque incisal margins and translucent shades, used mainly as a final cover layer or for enhancing incisal translucencies (e.g., between mamelons or between dentinal body and incisal margin). Naming is very often very confusing for the clinician while very often shades are called “dentin” or “enamel” while they could be used for their opacity characteristics despite the restoration portion they will be applied to [26]. It is very common to use an opaque material (often called “dentin”) in the incisal margin where on sound teeth there is enamel.

**Figure 58:**
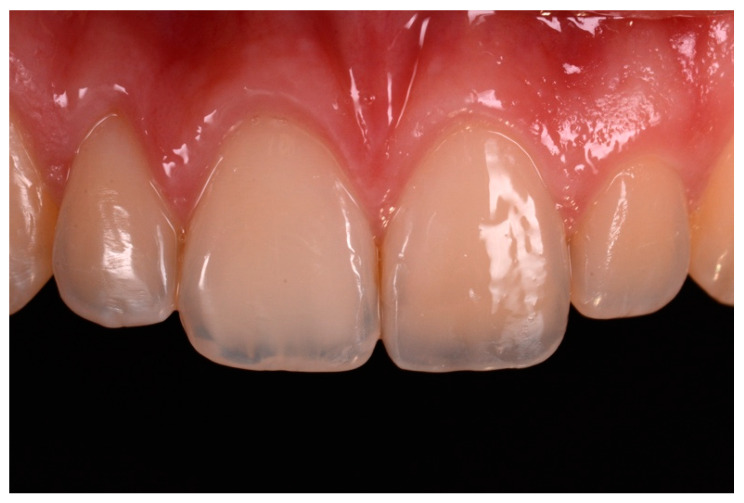
A more accurate management of the frame could have improved the thin and not festooned incisal opaque margin on left central incisor.

**Figure 59:**
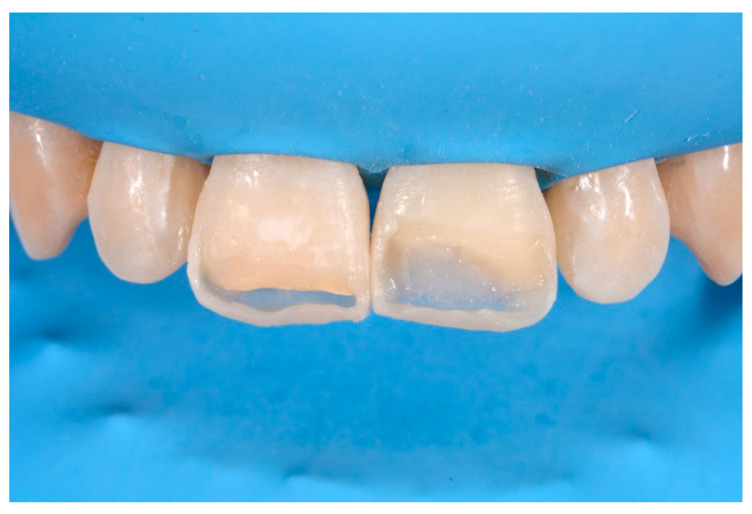
Completing the restorations without previously reducing the incisal frame could provide an unpleasant final esthetic outcome.

A wise balance of these opaque and translucent shades represents the key factor in obtaining reliable esthetic outcomes and great attention should be paid for their correct positioning [10,27]. Nevertheless, it happens that, during the restorative procedures, some inaccuracies could be made resulting in having opaque shades in place of translucent ones. This is generally realized too late, after finishing and polishing procedures thus compromising the final esthetic result [25]. In Figure 58 in fact the esthetic outcome of the left central incisor could have been improved if the opaque incisal margin had been made thicker and festooned. Conversely, we can notice from Figure 59 excessive thickness in the opaque incisal margin that, if not reduced, could provide an unpleasant final esthetic result.

In this article, a step-by-step procedure aimed at solving this issue is therefore proposed. There are several layering techniques proposed for anterior esthetic restorations. The one adopted from the authors of the present article is based on the completion of a frame before applying the dentinal body and finally the superficial outer enamel layer. The frame is defined by palatal wall, incisal margin, and interproximal wall/s. Despite the preliminary color and opacity chart can help the clinician in applying correct shades in proper place, some discrepancies in thickness, outline and position can occur. A too thick incisal opaque margin could for example affect the incisal third of a restoration compromising the incisal halo effect. Discrepancies in interproximal walls can also affect final result while they could reduce the volume to be left for dentinal anatomy. Not only the thickness of the abovementioned portions is important. The correct shape, height and emergence of the interproximal walls can provide the clinician many references to take advantage of in managing the thickness of the outer layer that is crucial for the value of the restorations as supported by several authors [28,29]. The modification of the frame could rise questions on how to treat the modified surface to add further composite and about the reliability of this procedure. Modifying a restoration and subsequently adding composite could be assimilated to an immediate composite repair procedure [30].

Composite repair is a reliable procedure that is generally carried out either immediately (shade adjustments, contact point augmentation or shape modification) or on aged composite restorations (in case of secondary caries or fracture) [31,32,33].

Dieckmann et al. [30] reported that the repair bond strength of fresh composite pretreated with diamond bur, silane application and adhesive did not show significative differences (65–75 MPa) from the positive control group (no repair). Significant differences were instead reported for repair in aged composite surfaces (30 MPa). The higher bond strength in immediate repair could be ascribed to the absence of degradation processes typical of aged composites that reduce the amounts of unsaturated double bonds [34] such as hygroscopic/stress expansion and hydrolytic degradation at the resin-filler interface [35,36]. The technique proposed in current article can be assimilated to an immediate repair and furthermore is performed in absolute isolation. Despite the above-mentioned difference between immediate and delayed repair bond strength, it must be remembered that lower values obtained in aged composites are in any case considered sufficient for adequate bonding [37,38]. This is also supported by clinical trials that have reported that repairs are equally clinically effective as total replacements [39,40]. These findings support our proposed technique while the addition of composite is performed few minutes before first curing cycles.

Regarding the surface treatment, the proposed technique involves the use of abrasive instruments (burs, discs, etc.) and do not advocate the use of AlO_2_ sandblasting. This is supported by Dieckmann et al. and by Kupiec KA and Barkmeier WW that reported no statistical differences in repair performed with or without sandblasting [30,41]. Regarding the diamond grit size, significant differences were observed by Valente et al. who reported higher bond strength when fine-grit (46 microns) diamond burs were used for repair in respect to coarse or extra-fine ones [42]. Conversely, no significant differences in grit size of diamond bur were reported by other authors [30,43].

In the proposed procedure, a coupling agent was employed for the following reasons: creation of covalent bonds of exposed fillers; copolymerization with methacrylate groups of repair material [44]; increase the wettability of the substrate and help the infiltration of bonding agent into the treated surface [45]. Furthermore, our approach is corroborated by several studies that reported improved composite repair bond strength if silane coupling agent was applied before the bonding agent [37,45,46,47,48]. The adhesive system used in the proposed technique is the well-studied and established Optibond FL (Kerr, Bioggio, Switzerland) [49,50,51]. The primer of the adhesive was also applied in the proposed procedure while Rathke et al. found significant higher bond strength in composite-to-composite repair performed with both components (primer and bonding) in respect to the application of bonding only [52].

The proposed technique, strictly applied in all cases, resulted in clinical acceptable results despite of the different composite used. This technique aims to allow the clinician to precisely manage opacities and translucencies of anterior composite restorations therefore could help the clinician in achieving reliable esthetic results. Among the limitations of the proposed technique there is the absence of clinical trials that could validate the procedure and the use of only one adhesive type (E&R three steps).

## 4. Conclusions

The proposed technique is an easy procedure aimed to reduce esthetic unpleasant results in direct anterior restorations. The precise execution of the frame and its eventual modification allows the clinician to get more predictable results in terms of shade and translucency management.

Modification of composite frame during layering procedures, can be, therefore, considered a safe and reliable approach to enhance the esthetic outcome of anterior composite restorations.

## Data Availability

Data sharing not applicable.
